# Supplementary material for: Efficacy and Safety of Pathogen-Reduced Platelets Compared with Standard Apheresis Platelets: A Systematic Review of RCTs
Source: Pathogens. 2022 Jun 1;11(6):639. doi: 10.3390/pathogens11060639 (PMC9231062; doi:10.3390/pathogens11060639)
Supplement: Supplementary file 1 [file pathogens-11-00639-s001.zip › pathogens-1745917-supplementary.pdf]

Data and analyses

1 Pathogen-reduced ptl. vs standard ptl.

Outcome or Subgroup	Studies	Participants	Statistical Method	Effect Estimate
1.1 Any bleeding events (grade 1-4): >7 d FU	7	1931	Risk Ratio (M-H, Random, 95% CI)	1.03 [0.85, 1.24]
1.1.1 intercept vs controls	6	1821	Risk Ratio (M-H, Random, 95% CI)	0.99 [0.80, 1.22]
1.1.2 mirasol vs controls	1	110	Risk Ratio (M-H, Random, 95% CI)	1.38 [0.95, 2.02]
1.2 Significant (grade ≥2) bleeding	9	3033	Risk Ratio (M-H, Random, 95% CI)	1.16 [1.02, 1.32]
1.2.1 intercept vs controls	5	1876	Risk Ratio (M-H, Random, 95% CI)	1.05 [0.95, 1.16]
1.2.2 mirasol vs controls	4	1157	Risk Ratio (M-H, Random, 95% CI)	1.39 [0.99, 1.96]
1.3 Severe (≥3) bleeding events	11	3297	Risk Ratio (M-H, Random, 95% CI)	1.09 [0.76, 1.56]
1.3.1 intercept vs controls	6	1979	Risk Ratio (M-H, Random, 95% CI)	1.15 [0.62, 2.13]
1.3.2 mirasol vs controls	4	1147	Risk Ratio (M-H, Random, 95% CI)	1.34 [0.74, 2.42]
1.3.3 theraflex vs controls	1	171	Risk Ratio (M-H, Random, 95% CI)	0.32 [0.01, 7.79]
1.4 No. pts with acute transfusion reactions	7	1780	Risk Ratio (M-H, Random, 95% CI)	0.95 [0.62, 1.47]
1.4.1 intercept vs controls	6	1764	Risk Ratio (M-H, Random, 95% CI)	0.95 [0.62, 1.47]
1.4.2 mirasol vs controls	1	16	Risk Ratio (M-H, Random, 95% CI)	Not estimable
1.5 Any adverse events	12	3445	Risk Ratio (M-H, Fixed, 95% CI)	1.09 [1.01, 1.19]
1.5.1 intercept vs controls	6	2017	Risk Ratio (M-H, Fixed, 95% CI)	1.01 [0.89, 1.15]
1.5.2 mirasol vs control	6	1428	Risk Ratio (M-H, Fixed, 95% CI)	1.16 [1.04, 1.30]

1.6 serious adverse events	13	3247	Risk Ratio (M-H, Fixed, 95% CI)	1.01 [0.82, 1.24]
1.6.1 intercept vs controls	7	2078	Risk Ratio (M-H, Fixed, 95% CI)	1.08 [0.86, 1.37]
1.6.2 mirasol vs controls	5	1027	Risk Ratio (M-H, Fixed, 95% CI)	0.78 [0.49, 1.25]
1.6.3 theraflex vs controls	1	142	Risk Ratio (M-H, Fixed, 95% CI)	Not estimable
1.7 1-hr CI	10	1846	Mean Difference (IV, Random, 95% CI)	-7.10 [-10.58, -3.62]
1.7.1 intercept vs controls	8	1479	Mean Difference (IV, Random, 95% CI)	-7.24 [-11.41, -3.06]
1.7.2 mirasol vs controls	1	196	Mean Difference (IV, Random, 95% CI)	-8.90 [-18.47, 0.67]
1.7.3 theraflex vs control	1	171	Mean Difference (IV, Random, 95% CI)	-5.01 [-8.55, -1.47]
1.8 1-hr CCI	11	1932	Mean Difference (IV, Random, 95% CI)	-3.15 [-4.30, -2.00]
1.8.1 intercept vs controls	8	1467	Mean Difference (IV, Random, 95% CI)	-2.97 [-4.47, -1.48]
1.8.2 mirasol vs controls	2	294	Mean Difference (IV, Random, 95% CI)	-4.12 [-6.29, -1.96]
1.8.3 theraflex vs control	1	171	Mean Difference (IV, Random, 95% CI)	-2.63 [-4.44, -0.82]
1.9 24-hrs CI	9	1800	Mean Difference (IV, Random, 95% CI)	-6.65 [-8.44, -4.86]
1.9.1 intercept vs controls	7	1435	Mean Difference (IV, Random, 95% CI)	-7.61 [-9.45, -5.77]
1.9.2 mirasol vs controls	1	194	Mean Difference (IV, Random, 95% CI)	-4.30 [-7.38, -1.22]
1.9.3 theraflex vs control	1	171	Mean Difference (IV, Random, 95% CI)	-3.84 [-7.06, -0.62]
1.10 24-hrs CCI	11	2435	Mean Difference (IV, Random, 95% CI)	-3.18 [-3.96, -2.41]
1.10.1 intercept vs controls	8	1960	Mean Difference (IV, Random, 95% CI)	-3.51 [-4.44, -2.58]
1.10.2 mirasol vs controls	2	304	Mean Difference (IV, Random, 95% CI)	-2.37 [-3.68, -1.06]

1.10.3 theraflex vs control	1	171	Mean Difference (IV, Random, 95% CI)	-2.08 [-3.84, -0.32]
1.11 No. individuals with ptl. refractoriness	10	2380	Risk Ratio (M-H, Fixed, 95% CI)	2.59 [1.98, 3.39]
1.11.1 intercept vs controls	6	1211	Risk Ratio (M-H, Fixed, 95% CI)	2.85 [1.96, 4.15]
1.11.2 mirasol vs controls	3	601	Risk Ratio (M-H, Fixed, 95% CI)	2.46 [1.61, 3.76]
1.11.3 theraflex vs controls	1	568	Risk Ratio (M-H, Fixed, 95% CI)	1.81 [0.71, 4.64]
1.12 No. individuals with ptl refractoriness and alloimmunisation	11	2628	Risk Ratio (M-H, Fixed, 95% CI)	1.77 [1.47, 2.13]
1.12.1 intercept vs controls	7	1390	Risk Ratio (M-H, Fixed, 95% CI)	1.61 [1.28, 2.02]
1.12.2 mirasol vs controls	3	670	Risk Ratio (M-H, Fixed, 95% CI)	2.14 [1.50, 3.07]
1.12.3 theraflex vs controls	1	568	Risk Ratio (M-H, Fixed, 95% CI)	1.77 [0.74, 4.24]
1.13 No. Ptl transfusion/participant	9	2193	Mean Difference (IV, Fixed, 95% CI)	1.04 [0.84, 1.24]
1.13.1 intercept vs controls	6	1731	Mean Difference (IV, Fixed, 95% CI)	1.07 [0.85, 1.29]
1.13.2 mirasol vs controls	2	291	Mean Difference (IV, Fixed, 95% CI)	1.06 [0.26, 1.87]
1.13.3 theraflex vs control	1	171	Mean Difference (IV, Fixed, 95% CI)	0.73 [0.04, 1.42]
1.14 No. RBC transfusions/participant	9	2193	Mean Difference (IV, Fixed, 95% CI)	0.32 [0.14, 0.50]
1.14.1 intercept vs controls	6	1731	Mean Difference (IV, Fixed, 95% CI)	0.27 [0.08, 0.47]
1.14.2 mirasol vs controls	2	291	Mean Difference (IV, Fixed, 95% CI)	0.42 [-0.12, 0.96]
1.14.3 theraflex vs control	1	171	Mean Difference (IV, Fixed, 95% CI)	0.73 [0.04, 1.42]

1.15 Platelet transfusion interval (days)	11	2424	Mean Difference (IV, Random, 95% CI)	-0.22 [-0.41, -0.03]
1.15.1 intercept	7	1391	Mean Difference (IV, Random, 95% CI)	-0.33 [-0.57, -0.10]
1.15.2 mirasol	3	862	Mean Difference (IV, Random, 95% CI)	-0.11 [-0.30, 0.08]
1.15.3 theraflex	1	171	Mean Difference (IV, Random, 95% CI)	0.51 [-0.20, 1.22]
